# Correlates of immunity to Group A Streptococcus: a pathway to vaccine development

**DOI:** 10.1038/s41541-022-00593-8

**Published:** 2023-01-17

**Authors:** Hannah Frost, Jean-Louis Excler, Shiranee Sriskandan, Alma Fulurija

**Affiliations:** 1 Murdoch Children’s Research Institute, Melbourne, VIC Australia; 2 International Vaccine Institute, Seoul, Republic of Korea; 3 Department of Infectious Disease, Imperial College London, London, UK; 4 MRC Centre for Molecular Bacteriology & Infection, Imperial College London, London, UK; 5 Telethon Kid’s Institute, Perth, WA Australia; 6 The University of Western Australia, Perth, WA Australia

**Keywords:** Translational research, Bacterial infection

## Abstract

Understanding immunity in humans to Group A Streptococcus (Strep A) is critical for the development of successful vaccines to prevent the morbidity and mortality attributed to Strep A infections. Despite decades of effort, no licensed vaccine against Strep A exists and immune correlates of protection are lacking; a major impediment to vaccine development. In the absence of a vaccine, we can take cues from the development of natural immunity to Strep A in humans to identify immune correlates of protection. The age stratification of incidence of acute Strep A infections, peaking in young children and waning in early adulthood, coincides with the development of specific immune responses. Therefore, understanding the immune mechanisms involved in natural protection from acute Strep A infection is critical to identifying immune correlates to inform vaccine development. This perspective summarises the findings from natural infection studies, existing assays of immunity to Strep A, and highlights the gaps in knowledge to guide the development of Strep A vaccines and associated correlates of protection.

## Introduction

Group A Streptococcus (Strep A, *S. pyogenes*) is among the top 10 leading causes of global infection-related morbidity and mortality across a diverse clinical spectrum including acute infections such as pharyngitis and impetigo, invasive infections and immune-mediated sequelae including acute rheumatic fever, rheumatic heart disease (RHD) and acute glomerular nephritis^1,2^. Despite successful treatment with penicillin, Strep A disease control remains challenging, leaving a vaccine as the most effective option for disease prevention^3^. To date, no licensed vaccine against Strep A exists and there is a lack of understanding of the mechanisms of protective immunity. This is a significant impediment to vaccine development both in terms of identifying optimal antigenic targets of vaccination, and developing assays that act as correlates of protection (CoP), the latter being a major focus of current Strep A research^4^.

Importantly, once identified, CoP assays could replace the need for clinical endpoints in vaccine efficacy trials, reducing the requirement for lengthy and costly studies with the disease as an endpoint^5,6^. For Strep A, this could obviate the burden of quantifying Strep A pharyngitis in hard-to-reach populations and reduce the need to judge vaccine efficacy based on the incidence of RHD, an autoimmune sequela that may arise years after repeated Strep A infections. CoP assays that are readily transferable between laboratories and countries and use standardized reagents that do not require specialist culture conditions or know-how are more likely to be accepted by licensing authorities. Furthermore, such assays may also allow for ongoing surveillance of immunity in target populations. At present, there are several knowledge gaps hindering the development of such assays for Strep A^7^, but the gaps are closing.

## Natural immunity against Strep A

The strongest evidence of protective immune responses against Strep A is the observed decreased susceptibility to infection with increasing age^7^. The frequency of acute Strep A infections peaks in childhood, with a much lower incidence of these diseases in adulthood. By contrast, invasive infections are seen in both the very young and very old populations^8^. This evidence provides a rationale to believe that an effective vaccine against Strep A is achievable.

The incidence of symptomatic throat infections, including scarlet fever, increases significantly around 4 years of age^9^. This could be due to the expansion of tonsil tissue allowing greater access for Strep A, increased exposure to other children at the start of school, or simply an artefact of school-aged children being able to articulate throat pain. Towards the end of childhood, the frequency of strep sore throats diminishes markedly. A similar peak and fall in incidence occur with Strep A skin infections, at a slightly earlier age than throat infections^7^. Invasive infections, seen in both the very young and very old populations^10^, may be associated with immune system naivety and immunosenescence respectively, along with the increased risk of skin injury and exposed portals of entry.

## Immunity to primary infection

Non-invasive infections would be the ideal target of vaccination. The mechanisms that confer resistance to colonisation and primary infection of the oropharynx or skin are unknown, but likely include a combination of prevention of bacterial adhesion, innate defence mechanisms, opsonophagocytic killing of bacteria supported by antibody and complement, inhibition of directly acting virulence factors, inhibition of bacterial immune evasion strategies, and cellular immunity.

During streptococcal infections, there appears to be a temporal sequence of adherence and colonisation by the bacteria. The initial “pioneer” cells perform long-range adherence and form molecular bridges with host proteins^11^. The following “settler” cells have shorter-range adherence with higher affinity and specificity. As the bacterial “society” forms in biofilms, there is environmental sensing, extracellular polymeric substance formation and quorum sensing. Finally, a “community” is established with cell-to-cell signalling, coaggregation, metabolic synergy and genetic exchange^11^. It is unknown against which stage or stages of colonisation an effective immune response must act to inhibit the development of infection, or whether targeting one or more stages by vaccination will be required to prevent non-invasive colonisation or infection.

Animal models of nasopharyngeal infection have shown that whole bacteria, single and combinations of antigens, and passive immunisation can induce immunity^12,13^. The route of immunisation may also be important to induce an effective immune response against Strep A^14^. Intranasal vaccines can generate both secretory immunoglobulin A (IgA) at the mucosa and serum IgG, as well as cell-mediated forms of immunity that are less well characterised^15^. Mice are not naturally exposed to Strep A, so provide a naive experimental system. There are however several drawbacks to the use of mouse models in Strep A vaccine research, including the large inoculum required to establish infection, lack of tonsils, use of lethal intranasal models that can result in pneumonia, and use of adjuvants unsuitable for humans^16^.

**Fig. 1:**
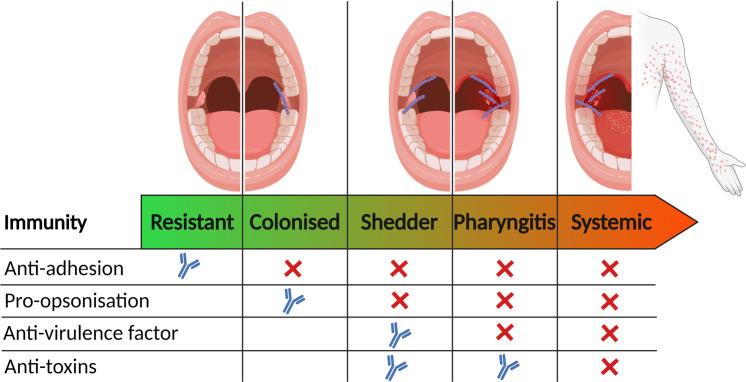
Proposed spectrum of antibody-derived immunity in oropharynx to explain the range of phenotypes observed in children^17^. In humans resistant to Strep A pharyngeal acquisition (first column), antibodies may inhibit adhesion and encourage opsonophagocytosis of bacteria before colonisation is established. In this situation antibodies targeting bacterial virulence factors and toxins may or may not be present. Colonised humans likely do not block adhesion with antibodies but, at least for some time, can limit the proliferation of bacteria. Shedders often have heavy colonisation but may inhibit symptomatic infection by controlling bacterial virulence factors. In most cases, pharyngitis is superficial and self-limiting, but can also develop into systemic illness including Scarlet fever (final column), which has been linked to toxins including superantigens^94^. The antibody symbols (blue) indicate where antibodies inhibit bacterial function, the crosses (red) where antibodies are lacking and no symbol where there is no requirement for antibodies. Created with Biorender.com.

There are many unknowns relating to human nasopharyngeal infection. When studying outbreaks of pharyngitis and scarlet fever in children, it was shown that over 25% of children acquire the outbreak strain^17^. Of these children the majority carry the bacteria asymptomatically, and some become heavy shedders of the strain, yet only a small number of children develop scarlet fever or pharyngitis^17^. Others never acquire the outbreak strain despite the same level of exposure. This may indicate a spectrum of different levels of immunity to different virulence factors in the human nasopharynx (Fig. 1), even in children.

## Immunity to invasive infection

Resistance to Strep A invasive infection is better understood, due to the uncommon ability of Strep A bacteria to grow in non-immune human blood. This forms the basis of the classic “Lancefield” assay^18^, which measures the opsonophagocytic capacity of donor plasma antibodies as a marker of resistance to invasive infection. Subcutaneous^19–21^, intramuscular^22,23^ and intranasal^24,25^ vaccination with adjuvanted protein vaccines appear effective at preventing even lethal invasive infections in mouse models of infection. Furthermore, passive transfer of immunity using antisera and pooled human intravenous immunoglobulin (IVIg) have all successfully induced immunity in animal models, particularly when highly concentrated^13^. IVIg contains high levels of anti-Strep A antibodies and is proposed as a clinical treatment for severe invasive Strep A disease^26,27^.

## Anti-Strep A antibodies

Although cell-mediated immunity no doubt contributes to long-term protection against Strep A, circulating IgG antibodies are recognised to be protective and are the most characterised adaptive immune mechanism. Furthermore, vaccines typically elicit antibody production and all CoP assays to date have relied upon antibody detection in vaccinated individuals; a better understanding of antibody responses is therefore central to vaccine development.

Reports of serum-based immune reactions to Strep A during Scarlet fever predate the consensus discovery of Strep A as the cause of Scarlet fever^28^. Convalescent human sera was shown to detoxify Strep A cultures and changed Dick skin test results from positive (susceptible) to negative (protected) following passive transfer^29^. Data from animal studies^30^, human serology^31^ and vaccine trials^32^ has linked antibody responses to specific Strep A antigens with various functions, including neutralisation of virulence factors, adhesins, and bacterial opsonophagocytosis.

The natural immunity to Strep A observed in adulthood is thought to be primarily driven by an accumulation of anti-Strep A antibodies^33,34^. Serosurveys of antibodies against the clinical serology antigens, Streptolysin O (SLO) and DNAse B, in different age groups and in different countries and socioeconomic settings support an age-related increase in these antibodies which are believed to represent biomarkers of Strep A exposure^35,36^. There are different theories for how Strep A antibodies confer protection: (1) natural human immunity to Strep A infection is type-specific and directed against the M-protein^37^, so that repeated exposures to different M types (or *emm*-clusters^38^) builds a repertoire of type-specific responses responsible for protection^39^; and (2) responses accumulate with repeated exposures to conserved (non-M-protein) Strep A antigens, raising the threshold of protection against subsequent infection^40,41^. Measurements of antigen-specific antibodies following Strep A infections indicate antibodies against both type-specific and conserved antigens occur concurrently and both likely contribute to immunity^42,43^.

Strep A has evolved several strategies to inhibit antibody function including the IgG-specific proteases, Ides and EndoS, that cleave antibodies at the hinge^44^ or Fc region^45^. The streptococcal cysteine protease, SpeB, regulates virulence by cleaving both host and bacterial proteins. Although SpeB is another example of a Strep A IgG cleaving protease^46^, it also inactivates bacterial-derived EndoS^47^. M and M-like proteins are an important family of Strep A virulence factors^48^. Their immune evasion functions include non-immune binding of IgG by the Fc region rendering them non-functional, and likely involvement in immune masking^49^.

Longitudinal studies suggest naturally acquired M protein antibodies may convey protection against infection, but not colonisation, and this may be strain-specific. Because colonisation without clinical symptoms (“asymptomatic carriage”) can be both immunogenic and immunologically silent, occur in the throat and skin, and can be of variable duration before resolution or resultant infection, here we describe pharyngeal acquisition events. Wannamaker et al. in 1953 followed 131 adult men with known baseline type-specific antibody responses and found the presence of type-specific antibody reduced subsequent symptomatic infection but not asymptomatic pharyngeal acquisition with the same M type^50^. Guirguis et al. also showed that the presence of type-specific bactericidal antibodies did not protect individuals from secondary pharyngeal acquisition, except against M1 strains^51^. Another longitudinal study found that 11 of the 75 pharyngeal acquisitions occurred in children who had pre-existing type-specific antibodies and only 12.5% of children developed type-specific antibodies following infection^43^. Thus, type-specific M protein-based immunity following infection is not guaranteed. In a longitudinal cohort of Fijian children, there was no association between the time to next Strep A skin infection and total serum IgG antibody titres to the M protein semi-conserved C-repeat region J8 peptide following primary skin infection^52^. It is important to note that vaccine-induced and natural immunity may differ significantly^23^.

## Existing assays of immunity to Strep A

Although protective immunity to Strep A is not well understood, the main mechanisms are likely to include the promotion of opsonophagocytosis, toxin or virulence factor neutralisation, and blocking of bacterial adhesion by B cell-derived antibodies and phagocytes, supported by T cells, cytokines, chemokines, and components of innate immunity. Several assays that mimic effector function in human immunity to Strep A are established as outlined below, although none would fulfil the specifications needed for a standardized, readily tranferrable assay of protection.

## Opsonophagocytosis assays

The Lancefield bactericidal assay, first described in 1927 by Todd, capitalised on the uncommon ability of Strep A bacteria to grow in blood^18^. The addition of test sera, heat-treated to remove endogenous complement proteins, to non-immune donor whole blood that is co-incubated with actively growing Strep A, measures the ability of test serum to inhibit the growth of the bacteria through promoting opsonophagocytic killing. The assay assumes that any reduction in Strep A growth is due to antibodies in the sera, which allows for a measure of functional immunity in sera^53^. However, the requirement to start with pre-screened, non-immune freshly drawn whole blood and freshly inoculated bacterial cultures, coupled with the overnight culture steps and colony counting make the assay laborious, complex and often poorly reproducible. Furthermore, the assay measures the net change over time in Strep A quantity, which is the consequence of both bacterial growth and opsonophagocytic killing. As such, it is not a pure measure of bacterial killing, and results are strain-specific.

There have been several attempts to provide a more reproducible opsonophagocytosis assay. A recent update to the assay protocol incorporates the Strep A virulence factor IdeS to digest endogenous antibodies in whole blood, removing the requirement of pre-screening for non-immune donors^54^. Other variations have utilised purified single donor human neutrophils co-incubated with different heat-treated donor sera, with flow cytometric analysis of bacterial uptake. Although such assays often do not quantify bacterial killing, the ability to measure the deposition of complement on the bacterial surface directly demonstrates the mechanism of opsonisation^55^ but is again limited by a need for freshly cultured bacteria, fresh donor blood, and neutrophil purification. A key recent refinement of these assays resulted in an HL-60 opsonophagocytic killing assay^56,57^, based on the established *S. pneumoniae* assay, using a cell line as the source of neutrophils, frozen bacterial stocks, and an endpoint that measures bacterial killing^58^. Although successfully applied to a few strain types, there remain several unknowns relating to the mechanism of uptake and subsequent killing in these assays, including the subtype of antibody required to promote bacterial killing, the failure of some Strep A strains to perform in such assays, and the variable importance of M protein and other Strep A antigens.

**Fig. 2:**
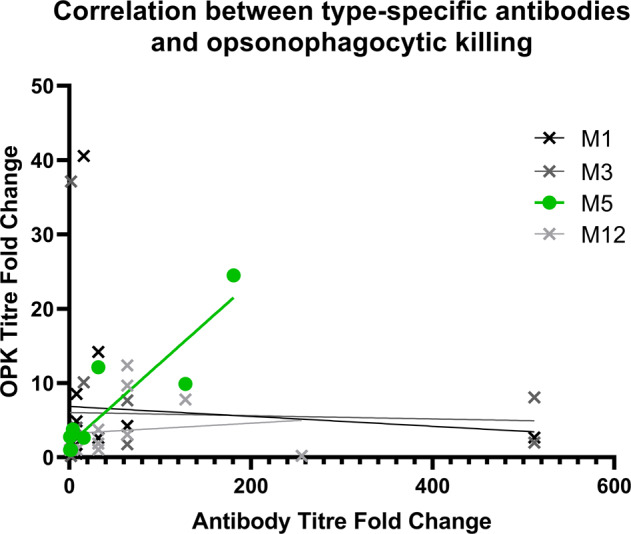
Correlation between titres of type-specific antibodies and opsonophagocytic killing (OPK) for four different Strep A strains following vaccination with the 30-valent vaccine candidate StreptAnova^TM^ that included M1, M3, M5, M12. A clear correlation is seen between OPK titre and antibody fold changes for M5 (green; *R*^2^ = 0.82), which is not apparent for the other strains. Data are from Pastural et al.^32^.

Opsonophagocytic assays may be a sound and plausible predictor of systemic protection in blood; however, such assays have not been definitively linked to protection against non-invasive infection in humans. In Wannamaker’s 1953 longitudinal study of the pharyngeal acquisition of Strep A, baseline bactericidal assays were able to predict the probability of developing a symptomatic infection, but they did not correlate with susceptibility to colonisation^50^. In studies where antibody functionality and titre data are available, any correlations appear to be individual, strain and/or disease specific. For example, in the recent phase 1 trial of a 30-valent Strep A vaccine, there was a good correlation between antibody titre against the M5 peptide and opsonophagocytic killing of this strain. By contrast, there was little to no correlation between antibody titre and the killing of three other strains studied (Fig. 2)^32^.

## Virulence factor neutralisation

The “Dick Test”, named after its inventors Dr. George and Dr. Gladys Dick, was a skin test for susceptibility to scarlet fever popular in the 1920s to 1950s^29^. The test involved intracutaneous injection of a small volume of filtered Strep A culture, which produced inflammatory reactions in susceptible individuals, and no reaction in those considered protected. The test represented a CoP assay of neutralising anti-superantigen activity which was also responsible for an alternative test using the “Shultz–Charlton phenomenon” whereby immune sera caused blanching of the scarlet fever-associated rash^59^. Unfortunately, the toxin-based vaccines assessed using these assays caused considerable inflammatory reactions and appeared to have reduced efficacy against other Strep A clinical syndromes^60–62^.

In recent decades, neutralising immunity to the superantigens associated with scarlet fever and toxic shock syndrome has been re-explored, partly to determine if the use of therapeutic IVIg might be of benefit. Building on the easily measured T-cell mitogenic effects of this group of toxins in vitro, titres of neutralising anti-toxin antibodies present in donor serum can be measured ex vivo. This demonstrated seroconversion and development of anti-SMEZ (Streptococcal mitogenic exotoxin) antibodies over a 7-day period in a patient who recovered from Streptococcal Toxic Shock Syndrome (STSS)^63^, and has also demonstrated the presence of anti-toxin antibodies in pooled IVIg^64,65^.

Established assays of immune responses to Strep A, often used to confirm a recent infection, include the measurement of functional neutralising anti-SLO and anti-DNAse B antibodies. Anti-SLO titres are calculated by the lysis of erythrocytes, as neutralising antibodies against SLO inhibit this function of the lysin. Likewise, anti-DNaseB titres are assessed based on inhibition of the degradation of DNA^66^. Most adults have some level of neutralising activity against these proteins indicative of previous Strep A exposure, but these antibodies are not known to be specifically protective. A rise in titres can indicate recent infection, although, in the case of SLO, it is now recognised that several strain types do not produce SLO owing to a variation in the promoter region^67^.

Several other virulence factors are known to influence Strep A pathogenesis and assays of immunity to these have been developed. One example is SpyCEP, a Strep A protease that can cleave CXC chemokines, particularly CXCL8 (IL-8)^68^. SpyCEP is a vaccine candidate and protection is believed to result from vaccine-induced antibodies that neutralise protease activity^69^; as such vaccine efficacy could not be evaluated using an opsonophagocytosis assay. Although the protein is very small (8 kDa) it is possible to demonstrate CXCL8 cleavage in vitro by purified SpyCEP, by gel electrophoresis or ELISA^68^, and to therefore inhibit this process using an immune serum. Such an assay could readily be adapted to detect vaccine-induced immunity to SpyCEP.

## Blocking adhesion

A sterilising immune response against Strep A must block the early stages of adhesion which lead to colonisation. Host-cell adhesion assays use monolayers of human cell lines, typically Detroit 562 to model pharyngeal colonisation and HaCat cells to model adhesion to the skin, and measure adherent live Strep A. Test serum containing antibodies against adhesins on the surface of Strep A can inhibit adhesion to these cells^70^. Animal studies have shown salivary IgA, but not serum IgG, against M protein reduces bacterial adherence to pharyngeal cells^71^. Rabbit antibodies against Strep A pili proteins inhibited bacterial adherence to a skin cell line^72^. As with antibodies that promote opsonophagocytosis and virulence factor neutralisation, there is little known about the characteristics of antibodies that inhibit adhesion, including isotype, glycosylation pattern, specific epitopes and affinity. As such, assays of immunity to combat adhesion are in their infancy yet may be of crucial functional importance.

## More tractable correlates of immunity

Finding a simpler, more reproducible method of detecting protective anti-Strep A antibodies would accelerate vaccine development. There are a number of Strep A antigens targeted by antibodies that either promote opsonophagocytosis^26,73,74^ or inhibit virulence factors^41,75–77^. Some of these antigens are included in candidate Strep A vaccines^19,78–80^. Detecting antibodies against these antigens is essential for vaccine clinical trials to demonstrate the magnitude and breadth of the antibody response in vaccinees. Measuring antibodies against additional non-vaccine antigens could differentiate vaccine-induced immunity from natural immunity. Such antibodies may be a marker of recent infection, and might have a further use diagnostically and in surveillance. Recently, bead-based assays that simultaneously measure antibody titres against three^81^, four^82^ and eight^83^ Strep A antigens from low-volume serum samples have been established. Such assays are agnostic to the function of the antibodies they detect; the purpose is to enable large scale screening using a readily transferrable assay that can be done in a few hours. The advantage of bead assays is the requirement for just a few microlitres of serum that can be tested against multiple antigens, which is of particular importance given the target Strep A vaccine population is children. Indeed, it is possible to adapt these assays to use antibody eluted from finger prick samples^83^, and potentially from mucosal fluids. Alternate technologies that deliver a similar outcome include mesoscale discovery (MSD) assays such as those employed during SARS-CoV-2 vaccine evaluation, which have been accepted as satisfactory and tractable immunogenicity assays by licensing authorities^84^.

## Future studies to develop CoP assays

Having developed reproducible, cell-free assays as above, in addition to the fundamental understanding provided by functional assays, it is now important to establish the best use of these assays and consider inclusion of standardised positive and negative controls that will be readily available to all those using the assays to facilitate comparison and correlation. At present, in the absence of any commercial vaccine, the best positive control is human pooled IVIg, while common negative controls include IgG-depleted serum and naive animal sera. Naive human sera, such as infant sera or cord blood, would be difficult to find and may be confounded by maternal antibodies.

Secondly, we need to understand whether the antibodies detected by such assays are functional or whether the antibodies simply correlate with functional immune activity. If either is found to correlate with clinical immunity this point may become less important. While both mechanistic and non-mechanistic CoPs are useful^85^, understanding mechanisms of immunity will have greater utility to vaccine development more broadly. Recent advances in systems serology have greatly improved the ability to link antigen-specific antibodies to effector functions^86^ and identify vaccine CoPs^87^. At present there is no gold standard for evaluation of immunity to Strep A, particularly if we accept that the classical Lancefield assay is a measure of immunity in blood but not the oropharynx. Unlike some viral vaccines, bacterial vaccines may employ several immune mechanisms, and it may be that no single functional assay can replicate the protection seen in humans^5^.

Finally, we need to optimise the assays to detect antibodies from different types of samples, including oral fluids, soft tissues and finger prick blood. Despite the oropharynx being a major site of Strep A infection, remarkably little is known about Strep A specific immunity in this mucosal tissue.

To understand how natural immunity develops we can look for signals in prospective cohort studies of children with active surveillance for Strep A infections^17,88^. Assays that measure antibody levels against Strep A antigens in oral fluid, specifically IgA, will be required to understand mucosal immunity. Studying adults as well as children, and conducting longitudinal studies, will provide further insight into what happens when a human encounters Strep A for the first time and how immunity evolves through adulthood. The CHIVAS-M75 human challenge model, where healthy adults were infected with an M75 Strep A, provides important information for the acute stage of colonisation and infection; most participants developed sore throat, but some did not^89^. The experiment paves the way to future studies that compare antibody profiles following vaccination, and in those who were resistant to the challenge and those who were susceptible. Similarly, the finding that young children, exposed to the same dose of Strep A exhibit a range of clinical phenotypes provides a natural opportunity to identify immunological differences between these phenotypes^17^. The roles of cellular immunity^90^, including T and B lymphocytes and their effectors, and cells in the tonsils^91^ warrant further investigation, as do genetic determinants of susceptibility and the differences between intranasal^92^ and intramuscular^93^ vaccine-induced immunity.

## Conclusions

Natural immunity to Strep A exists and it is likely that several immune mechanisms contribute to the protection against Strep A infection afforded to adults. With prospective surveillance of Strep A infections and high-quality sample collections it will be possible to identify immune correlates of natural protection. However, it would be exceedingly difficult to define a CoP for a vaccine without first demonstrating clinical protection in a reproducible, verifiable manner from vaccine trials. Nonetheless, with multiple vaccines planning to enter clinical trials and vaccine-challenge trials in the next few years, the potential to demonstrate protective efficacy seems closer. By combining known features of natural immunity, currently available assays of immunity and emerging findings from human trials, a CoP for Strep A is highly plausible.

## Data Availability

No new data are presented in this report. Data used to create Fig. 2 are from Pastural et al.^32^.
